# Epigenetic reprogramming of melanoma cells by vitamin C treatment

**DOI:** 10.1186/s13148-015-0087-z

**Published:** 2015-04-29

**Authors:** Christopher B Gustafson, Cuixia Yang, Kevin M Dickson, Hongwei Shao, Derek Van Booven, J William Harbour, Zhao-Jun Liu, Gaofeng Wang

**Affiliations:** John P. Hussman Institute for Human Genomics, Dr. John T. Macdonald Foundation Department of Human Genetics, University of Miami Miller School of Medicine, Miami, FL 33136 USA; Department of Surgery, University of Miami Miller School of Medicine, Miami, FL 33136 USA; Sylvester Comprehensive Cancer Center, University of Miami Miller School of Medicine, Miami, FL 33136 USA; Bascom Palmer Eye Institute, University of Miami Miller School of Medicine, Miami, FL 33136 USA; Department of Molecular Biology Laboratory, Shanghai Sixth People’s Hospital, Shanghai Jiaotong University, Shanghai, 200233 China

**Keywords:** Vitamin C, 5-hydroxymethycytosine, Epigenetic reprogramming, RNA-seq, Melanoma

## Abstract

**Background:**

The loss of 5-hydroxymethylcytosine (5hmC) has been identified as a novel epigenetic hallmark for melanoma. One of the known mechanisms underlying the loss of 5hmC is the decrease in expression of ten-eleven translocation family dioxygenase (TET) genes, which encode enzymes that catalyze the generation of 5hmC. Overexpressing TET2 was shown to partially reestablish a normal 5hmC profile in melanoma and decrease invasiveness in rodents. However, the feasibility to overexpress TETs in patients remains unclear. We and others have recently demonstrated that TETs require vitamin C as a cofactor to generate 5hmC. This finding prompted us to test whether vitamin C, as an alternative to overexpressing TETs, could rebuild 5hmC content in melanoma.

**Results:**

Consistent with previous reports, we found that the expression of TETs was decreased in various melanoma cell lines. In contrast, the expressions of sodium-dependent vitamin C transporters (SVCTs) were down-regulated in cell lines derived from melanoma metastases. Treatment of vitamin C at the physiological level (0.1 mM) promoted the content of 5hmC in melanoma cell lines derived from different stages toward the level of healthy melanocytes, which was comparable to the effect of overexpressing TET2. Vitamin C treatment decreased the malignancy of metastatic A2058 cells by inhibiting migration and anchorage-independent growth, while not exerting any effect on the rate of proliferation. Further, vitamin C treatment caused alterations in genome-wide transcriptions shown by RNA-seq, predominantly in ArhGAP30 and genes involved in extracellular matrix remodeling, which could underlie the decreased malignant phenotypes.

**Conclusions:**

Our data support the idea that vitamin C treatment increases 5hmC content in melanoma cells, while causing a decrease in tumor-cell invasiveness and clonogenic growth in soft agar. Thus, vitamin C could be a potential epigenetic treatment for melanoma.

## Background

Aberrant epigenetic alterations, along with genetic mutations, are known to contribute to the onset of cancer. Recently, the loss of 5-hydroxymethylcytosine (5hmC) has been identified as a novel hallmark for most, if not all, types of cancer (including melanoma) [1-4]. The content of 5hmC is relatively high in healthy melanocytes but is gradually lost during the progression from benign nevi through advancing stages of primary and metastatic melanoma [3]. 5hmC is converted from 5-methylcytosine (5mC), the major epigenetic modification of mammalian DNA, by a group of enzymes termed TET (ten-eleven translocation) family dioxygenases [5,6]. TETs can further oxidize 5hmC to 5-formylcytosine and 5-carboxylcytosine, which are eventually replaced by unmodified cytosine thus completing the process of demethylation. The cascade oxidation of 5mC by TETs and the ensuing base excision repair has been recognized as the most consistent mechanism underlying the active demethylation of DNA [7].

The loss of 5hmC demonstrates an epigenetic drift in the dynamics of DNA methylation-demethylation, which could potentially be involved in the pathogenesis of melanoma. Interestingly, 5hmC is relatively stable and is distributed with unique patterns in the genome [8,9]. Further, 5hmC recruits different sets of binding proteins, as compared to 5mC, and correlates with unique transcription profiles [10-12]. In addition to being a DNA demethylation intermediate, 5hmC serves as an epigenetic mark with unique regulatory capabilities. Therefore, the global loss of 5hmC can change genome stability and genome-wide transcription patterns, which ultimately leads to a cascade of phenotypic transformations from healthy melanocytes toward malignant melanoma.

One of the known mechanisms underlying the loss of 5hmC in melanoma is a lower expression of the TET and isocitrate dehydrogenases (IDH) genes [3,4], which encode enzymes that catalyze the production of 2-oxoglutarate, a co-substrate for the TET enzymes. It has been shown that overexpressing TET2 could partially reestablish a normal 5hmC profile in cultured melanoma cells and decrease their invasiveness in modeled animals [3]. These findings suggest that rebuilding the 5hmC content can be a potential treatment for melanoma. However, the feasibility to clinically overexpress TETs or IDHs in patients suffering from melanoma remains unclear.

TETs belong to the iron and 2-oxoglutarate-dependent dioxygenase superfamily. These dioxygenases utilize Fe^2+^ as a cofactor, 2-oxoglutarate as a co-substrate, and some of them require vitamin C (ascorbate) as an additional cofactor to achieve full catalytic activity. We and other groups have recently shown that vitamin C induces the generation of 5hmC, most likely by acting as a cofactor for TET dioxygenases to hydroxylate 5mC. Vitamin C at physiological levels has been shown to increase the global content of 5hmC *in vivo* and *in vitro* [13-17]. This previously unknown function of vitamin C in modulating DNA demethylation prompted us to test whether vitamin C, as an alternative to overexpressing TETs or IDHs, could be a potential epigenetic treatment for melanoma by rebuilding 5hmC profiles.

## Results

### The expression of TETs was decreased in melanoma cell lines

**Figure 1:**
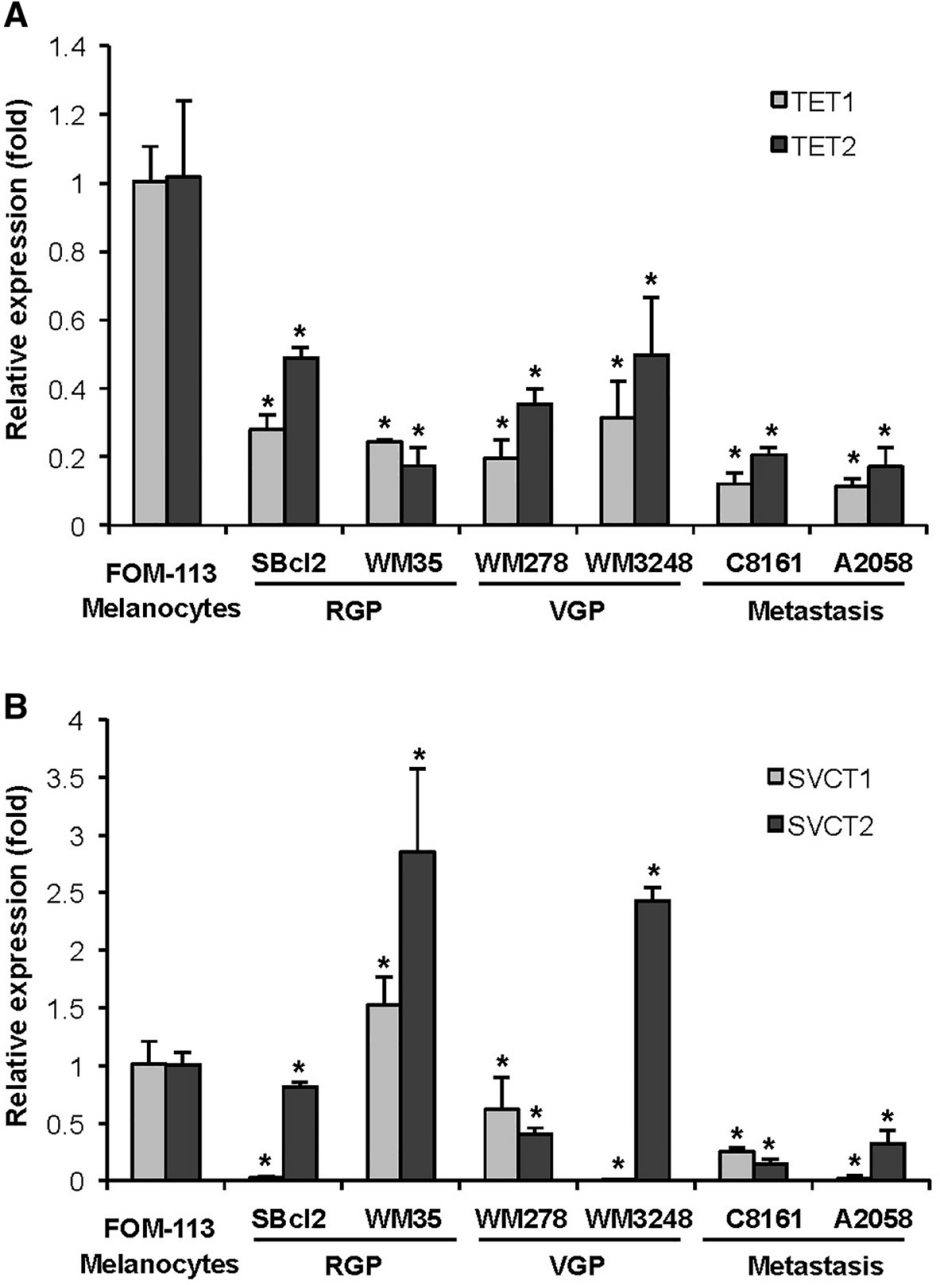
The relative transcription levels of TETs and SVCTs in melanoma cells. Levels of TETs and SVCTs (mean) in a healthy melanocyte line are set up as ‘1’. **(A)** The mRNA levels of TET1 and TET2, measured by qRT-PCR, are decreased in melanoma cell lines derived from RGP, VGP, and metastatic stages compared to a healthy melanocyte line FOM-113. **(B)** The mRNA levels of SVCTs are either decreased or increased in the examined melanoma cell lines. Comparatively, the transcription levels of both SVCTs are decreased in the melanoma cell lines (C8161, A2058) derived from the metastatic stage. (Mean ± SD, *P < 0.05).

We first evaluated the expression level of TETs (TET1, TET2, and TET3) in primary cultured healthy human melanocyte lines (FOM-113) and various melanoma cell lines using qRT-PCR. These melanoma cell lines include those that are derived from the radial growth phase (RGP, SBcl2 and WM35), vertical growth phase (VGP, WM278 and WM3248) and metastatic stage (C8161, A2058). Consistent with a previous report [3], we found that the expression of TET1 and TET2 was consistently decreased in all melanoma cell lines examined, as compared to the normal melanocyte line (Figure 1A). TET3 was not detectable by qRT-PCR in our lab. Of the cell lines evaluated, the lowest expression level of both TET1 and TET2 was observed in two metastatic melanoma cell lines.

### The expression of SVCT2 was decreased in metastatic melanoma cell lines

The uptake and accumulation of vitamin C by cells are mainly *via* sodium-dependent vitamin C transporters (SVCT). There are two types of SVCTs, which are, SVCT1 and SVCT2. We then measured the expression of SVCT1 and SVCT2 in the same melanoma cell-line panel used for the evaluation of TETs. Both SVCT1 and SVCT2 were detectable in these cell lines by qRT-PCR, although the expression level of SVCT1 is much lower than that of SVCT2 (approximately 3 cycles in difference). The mRNA levels of SVCT1 and SVCT2 were either decreased or increased in the examined melanoma cell lines compared to the normal melanocytes. However, only in those metastatic melanoma cell lines (C8161 and A2058), the expression of both SVCT1 and SVCT2 was consistently decreased, as compared to the healthy melanocytes (Figure 1B). Interestingly, it has been shown that the uptake rate of ascorbate (the dominant form of vitamin C in the plasma) by melanoma cells (SK-MEL-131) is only approximately 50% of the uptake rate by healthy melanocytes [18]. A decreased expression of SVCTs presumably could also result in a lower efficiency in vitamin C uptake in these metastatic melanoma cell lines. These results suggest that a local vitamin C deficiency, along with a decreased level of TETs, may contribute to the global loss of 5hmC in metastatic melanoma cells.

### Vitamin C treatment promoted 5hmC content in melanoma cells toward that of healthy melanocytes

**Figure 2:**
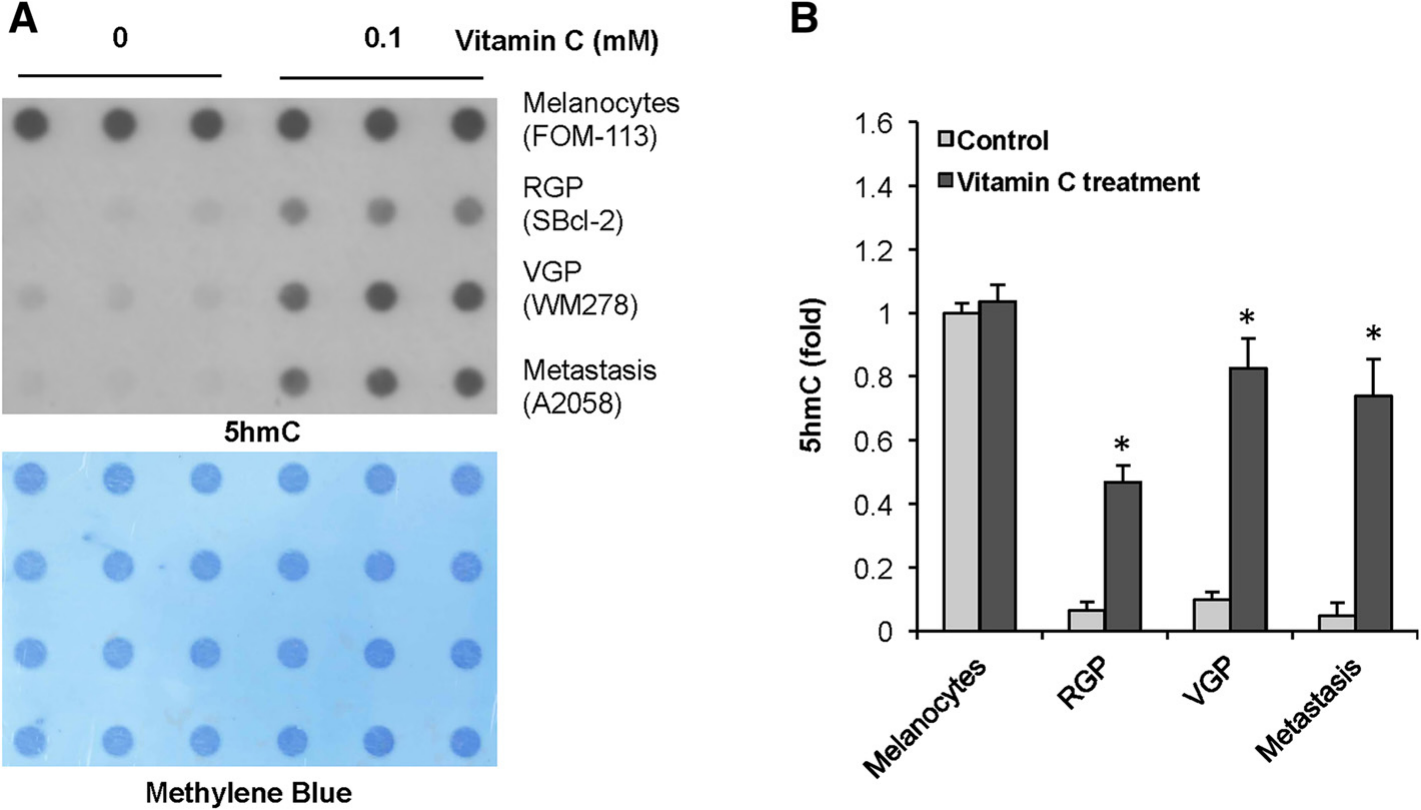
Vitamin C treatment raises 5hmC content toward that in healthy melanocytes. **(A)** The dot blot shows vitamin C treatment (0.1 mM) for 48 h and increases the global content of 5hmC in melanoma cells at different stages (RGP, VGP, and metastatic) toward the level in healthy melanocytes. **(B)** The semi-quantitative analysis of the dot-blot indicates that vitamin C significantly promotes 5hmC generation in melanoma cells. (Mean ± SD, *P < 0.01). The level of 5hmC (mean) in untreated melanocytes is set up as ‘1’. The relative levels of 5hmC in melanoma cells with or without vitamin C treatment are shown.

Next, we tested whether vitamin C treatment could increase the global content of 5hmC in melanoma cells. The content of 5hmC was relatively high in healthy melanocytes (FOM-113) but was barely detectable by dot-blot assay in SBcl-2 (RGP), WM278 (VGP), and A2058 (metastasis) melanoma cells cultured in a vitamin C-free medium. These cell lines were chosen based on their decreased expression of both SVCT1 and SVCT2. The average concentration of vitamin C in the plasma of humans is at approximately 0.05 mM range and could reach concentrations of up to approximately 0.15 mM [19]. We reasoned that a relatively higher concentration of vitamin C, but still in the range of physiological concentration, could compensate for the down-regulated SVCTs in melanoma cells. In this case, vitamin C at 0.1 mM was chosen for treatment. There was no obvious change in the content of 5hmC in healthy melanocytes after treatment with vitamin C (0.1 mM) for 48 h, which could be explained by the possible saturation of dot-blot signals. In contrast, vitamin C treatment dramatically increased the content of 5hmC in all three melanoma cell lines toward the level in healthy melanocytes (Figure 2). After treatment with vitamin C, the content of 5hmC in WM278 cells and A2058 cells was increased to approximately 80% of control melanocytes, while the content of 5hmC in SBcl-2 cells was only increased to approximately 50% of control melanocytes. It remains unknown to us why the response of SBcl-2 cells was less than the other cell lines, though the expression of TETs and SVCTs in SBcl-2 cells of RGP was at similar levels as the other melanoma cell lines. Nevertheless, these results suggest that vitamin C at physiological levels indeed promotes the content of 5hmC toward the level of healthy melanocytes, which is comparable to the effect of overexpressing TET2, as shown previously [3].

### Vitamin C time-dependently increased 5hmC content in A2058 cells

**Figure 3:**
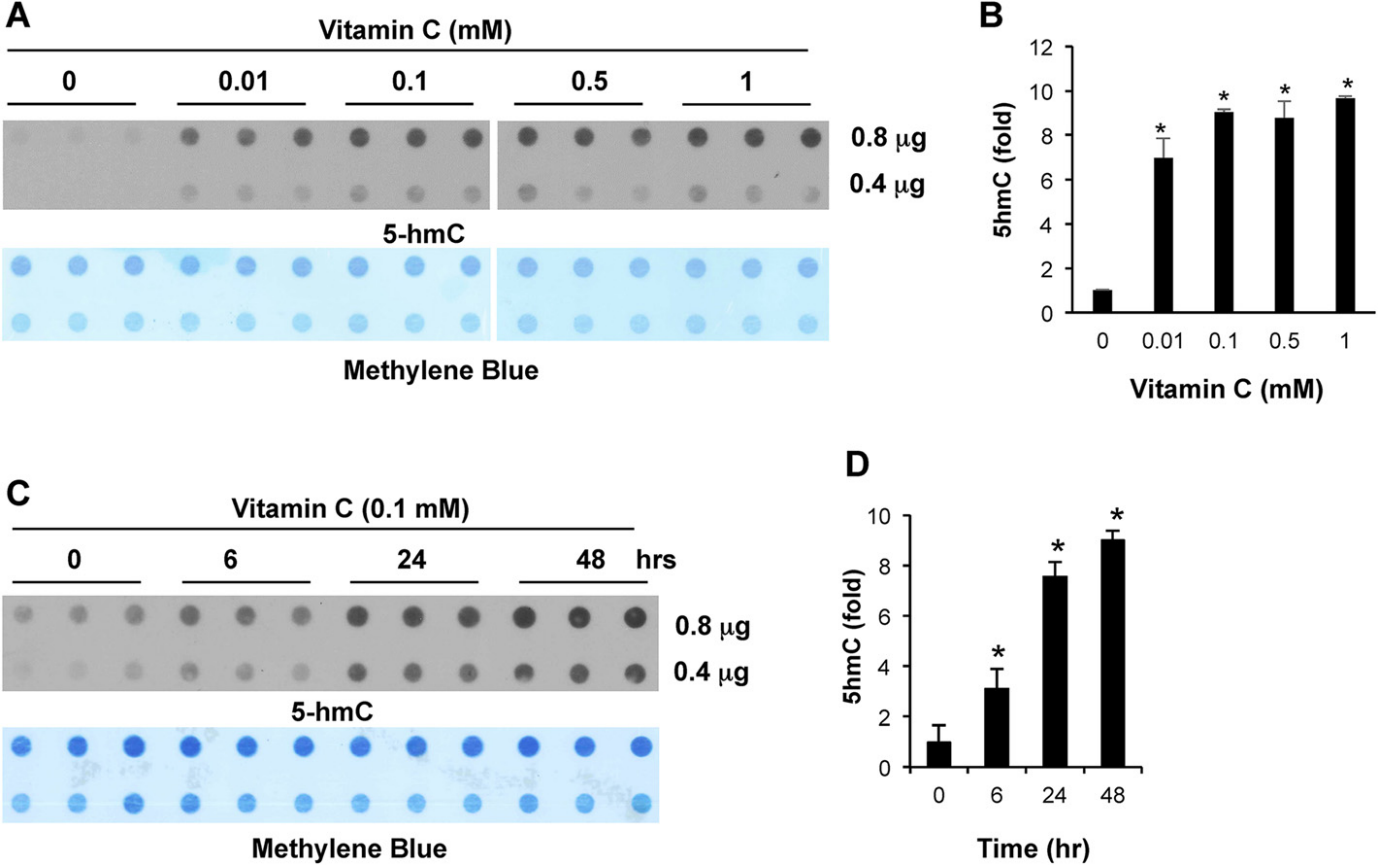
Vitamin C treatment increases the content of 5hmC in A2058 melanoma cells. **(A to B)** The dot-blot and semi-quantitative analysis of the dot-blot show that treatment with vitamin C (0.01 mM) increase 5hmC to approximately sixfold of the basal level. Higher concentrations of vitamin C (0.1 mM to 1 mM) induce 5hmC generation at a similar level (approximately ninefold in increase). **(C to D)** Vitamin C treatment (0.1 mM) time-dependently increases the content of 5hmC in A2058 cells as showed by the dot-blot and the semi-quantitative analysis of the dot-blot. Quantitative data are relative amounts of 5hmC.

Previous studies have implicated a potential pharmacological effect of elevating vitamin C to higher concentrations in the plasma (>0.3 mM) on certain types of cancer [20]. Pharmacological levels of vitamin C in the plasma can only be achieved through intravenous injections. Although intravenous injections in a certain way may complicate the delivery of vitamin C to patients, we still tested whether vitamin C at pharmacological levels has an even stronger effect on 5hmC generation in melanoma cells. The A2058 cell line was used as a cell model in the first published study on 5hmC reestablishment in melanoma. Following this, we chose the A2058 cell line as a model also considering their decreased expression of TETs and SVCTs. As described above, the 5hmC signal was barely detectable in A2058 cells when cultured without vitamin C. After supplementation of vitamin C (0.01 mM) in the medium for 24 h, we found that the content of 5hmC increased about sixfold, and 0.1 mM vitamin C induced further increase in 5hmC (nearly ninefold), as compared to the basal level (Figure 3A & B). However, 5hmC was induced to a similar level (*P* > 0.05) in response to vitamin C treatment, regardless of how low (0.1 mM) or high (0.5 mM and 1 mM) the concentrations were. This suggests that vitamin C at pharmacological levels does not incur greater benefits in promoting 5hmC generation in melanoma cells, as compared to vitamin C at physiological levels.

Similar to what we previously observed in non-malignant cells [13], vitamin C treatment also enhanced the generation of 5hmC in A2058 cells in a time-dependent manner. The 5hmC content was increased approximately threefold in 6 h, approximately eightfold in 24 h, and approximately ninefold in a 48-h post vitamin C treatment (0.1 mM) (Figure 3C & D). This result suggests that TET enzymes, though at a lower level than in healthy melanocytes, are ready to generate 5hmC in melanoma cells but are waiting for vitamin C to assist the hydroxylation reaction. Thus, the accumulated vitamin C within the cells led to a time-dependent increase of 5hmC. It is important to note that the difference between the time points of 24 h and 48 h was miniscule, which could in part be due to the fact that 5hmC is not maintained during DNA replication in these rapidly growing cells. These results once again indicate that vitamin C at physiological levels partially reestablishes the content of 5hmC in melanoma cells, which is comparable to the effect of overexpressing TET2 in the same cell line [3].

### Vitamin C treatment inhibited the malignant phenotype of melanoma cells

**Figure 4:**
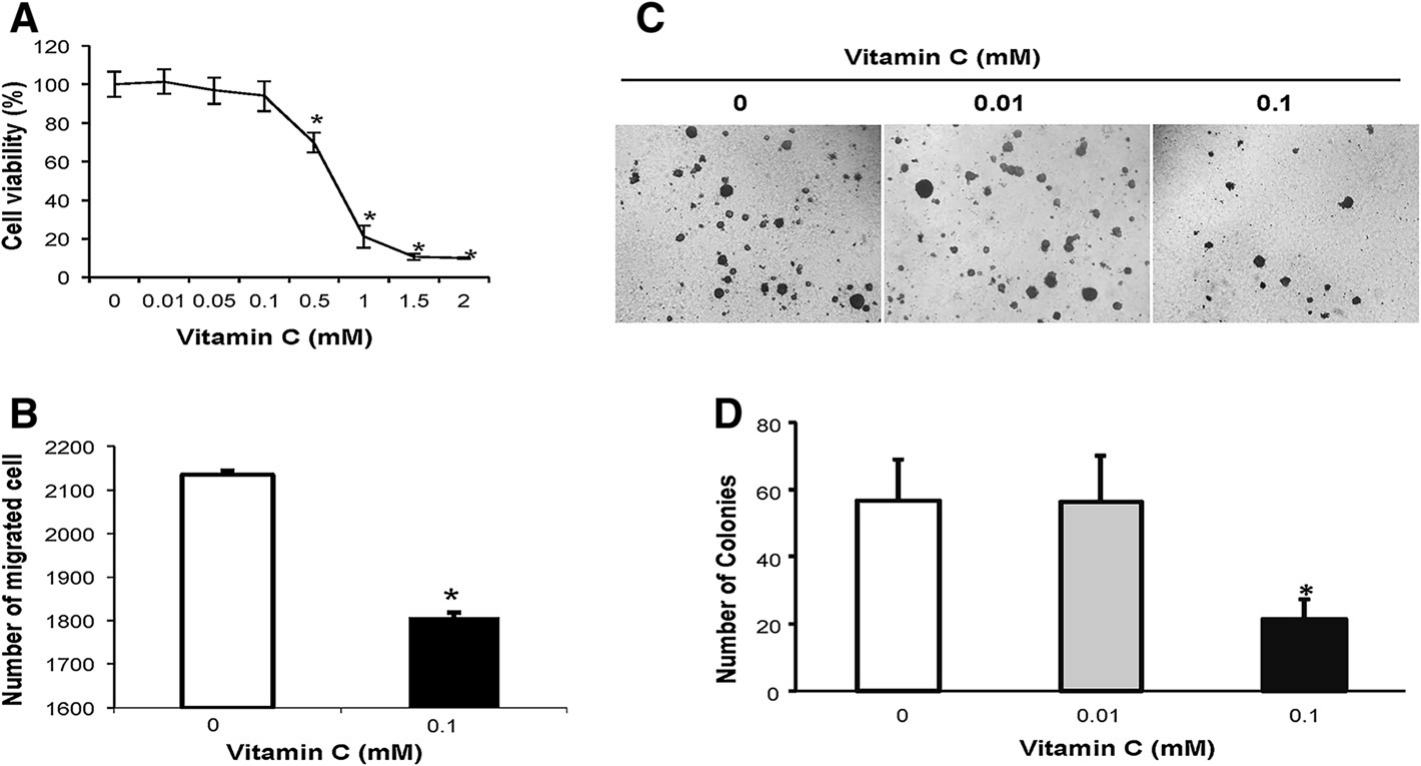
Vitamin C treatment decreases the malignant phenotype of A2058 cells *in vitro*. **(A)** MTT assay shows that Vitamin C at pharmacological levels (0.5 mM to 2 mM) inhibits A2058 cell proliferation while it does not affect A2058 cell proliferation at physiological levels (0.05 mM to 0.1 mM). **(B)** Treatment with vitamin C (0.1 mM) mitigates A2058 cell migration as tested by the transwell assay. **(C to D)** Vitamin C treatment (0.1 mM) inhibits the anchorage-independent growth of A2058 cells, and 0.01 mM vitamin C has no obvious effect. A representative image of soft-agar assay is shown in **(C)**. Quantitative data of colonies formed (#/well) in the soft-agar assay **(D)**. (*P < 0.05).

We then questioned what the functional consequences of the vitamin C-increased 5hmC content could be in terms of melanoma malignancy. According to our observation described above, vitamin C at physiological concentrations is able to restore the global content of 5hmC as efficiently as to that of pharmacological concentrations in melanoma cells *in vitro*. In that regard, we tested the effect of vitamin C at physiological concentrations on behaviors of melanoma cells. In the cell proliferation analysis, we observed that treatment with vitamin C at physiological concentrations (0.05 mM to 0.1 mM) did not evidently affect the growth rate of A2058 cells (Figure 4A) as well as healthy melanocytes (data not shown). In contrast, vitamin C at pharmacological concentrations (0.5 mM to 2 mM) significantly inhibited not only A2058 cell proliferation (*P* < 0.05), but also the cell growth rate of healthy melanocytes (data not shown). Since such high concentrations (0.5 mM to 2 mM) of vitamin C treatment appeared to be toxic to many types of non-malignant cells including healthy melanocytes, pharmacological concentration of vitamin C treatment might not be a practicable option for cancer therapy. This might explain why vitamin C treatment at pharmacological concentrations resulted in inconsistent therapeutic results for cancer [20]. Overall, our results suggest that vitamin C at physiological concentrations does not affect cell proliferation of both melanoma (A2058) cells and healthy melanocytes. It appears to be safe for clinical application.

Interestingly, although vitamin C at physiological concentrations had little effect on cell proliferation of melanoma (A2058) cells, it indeed altered melanoma cell migration and *in vitro* transformation. In our experiments using the transwell cell migration assay to evaluate the effect of vitamin C treatment at physiological concentrations on melanoma cell migration, we treated A2058 cells with or without vitamin C (0.1 mM) for 24 h and tested their ability to migrate in the transwell. We observed that vitamin C at a physiological level (0.1 mM) significantly inhibited A2058 cell transwell migration (Figure 4B).

Anchorage-independent growth is one of the hallmarks of transformation. It is considered the most accurate and stringent *in vitro* assay to detect malignant transformation of cells *in vitro*. We further examined the effect of vitamin C treatment at physiological concentrations on anchorage-independent growth of melanoma cells. We observed that supplementation of vitamin C (0.1 mM) drastically inhibited A2058 cell colony formation in the soft agar, while 0.01 mM vitamin C did not obviously affect the colony formation (Figures 4C & D). This result suggests that vitamin C at a physiological level (0.1 mM) mitigated malignant transformation of melanoma cells. Considered together, our results demonstrate that vitamin C at physiological concentrations could significantly reduce the malignant phenotype of A2058 cells *in vitro* by inhibiting cell migration and transformation but not suppressing cell proliferation. Hence, vitamin C at physiological concentrations might be applied as a safe and clinically sound therapeutic option.

### Vitamin C treatment shifted the transcriptome of melanoma cells

We examined the possible mechanisms that could reveal how vitamin C suppresses cell migration and anchorage-independent growth of A2058 cells. Vitamin C is a micronutrient and has been proven to serve many different functions, including anti-oxidation and acting as a cofactor for a list of iron and 2-oxoglutarate-dependent dioxygenases. We and others have shown that the effect of vitamin C on 5hmC generation is independent of its property as a general reducer [13-17]. Of the known enzymes requiring vitamin C as a cofactor, only TETs and its mediated hydroxylation of 5mC to 5hmC have known direct impacts on gene transcriptions. Thus, we hypothesized that the vitamin C-induced global increase of 5hmC could, at least in part, be responsible for the phenotypic consequences of A2058 cells by changing gene expression profiles.

**Figure 5:**
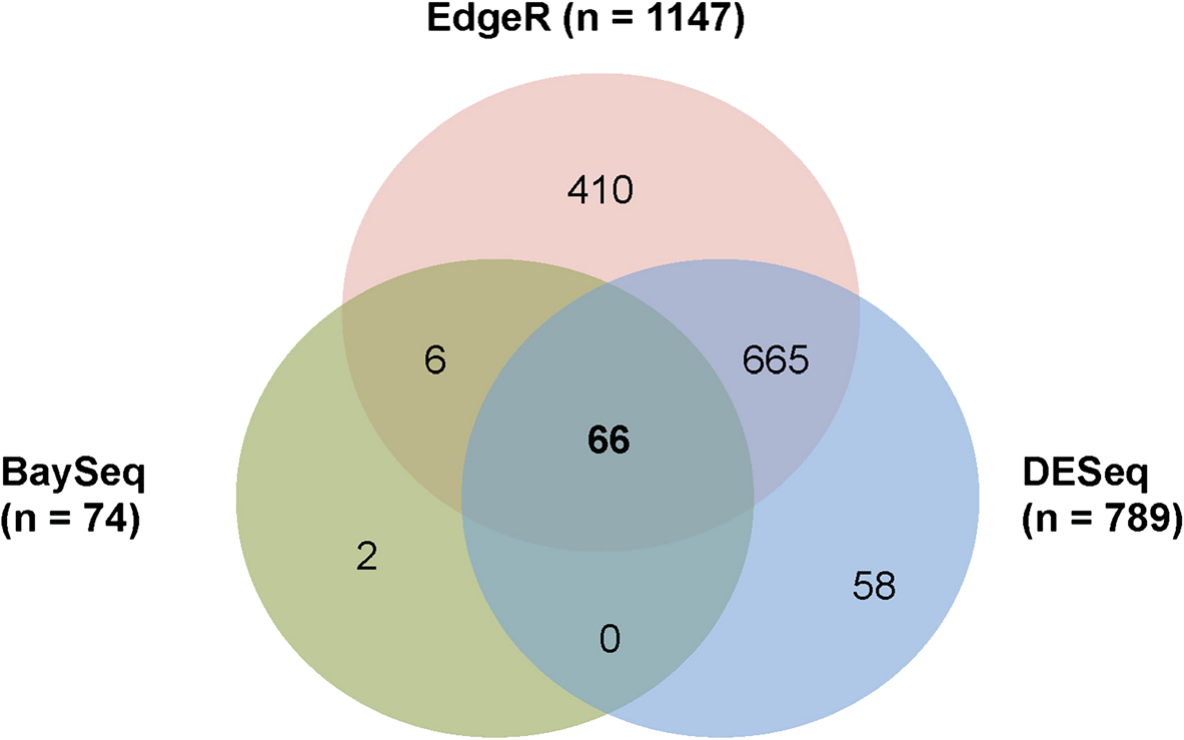
Vitamin C treatment shifts the transcriptome of A2058 cells. After vitamin C (0.1 mM) treatment for 48 h, differentially expressed genes are identified by EdgeR (1,147 genes), DEseq (789 genes), and Bayseq (74 genes) analysis. However, only 66 genes are overlapped by the analyses of all three methods.

To test our hypothesis, we conducted RNA-seq to evaluate the influence of vitamin C treatment on the transcriptome of A2058 cells. In order to minimize potential secondary effects in the long term, A2058 cells were treated with vitamin C at physiological concentrations (0.1 mM) for 48 h. DNA and RNA were simultaneously extracted from A2058 cells cultured in the same wells (*n* = 3 per group). After the vitamin C-induced 5hmC were confirmed by dot-blot assay (data not shown), RNA were submitted to the Sequencing Core of John P. Hussman Institute of Human Genomics at the University of Miami. We applied rigorous bioinformatic and statistical approaches to analyze the RNA-seq data. The results showed that 1,147 genes were differentially expressed by EdgeR, 789 genes by DEseq, and 74 genes by Bayseq, respectively. However, only 66 genes including a few of non-coding transcripts were significantly and differentially expressed in the analyses of all three methods - EdgeR, DESeq, and BaySeq (Figure 5). Of the 66 genes, seven genes were down-regulated while the rest of the 59 genes were up-regulated. Then, we chose the top ten genes for validation based on fold changes (≥1.6-fold increase or ≤0.7 fold decrease) in multiple callers. The expression changes of all ten genes had been verified by qRT-PCR (Table 1). However, the ten verified genes may not be able to fully explain the effects of vitamin C on the altered malignant phenotype of A2058 cells. The most significant change induced by vitamin C treatment occurs in the gene rho GTPase activating protein 30 (ARHGAP30, ≥tenfold increase). Unfortunately, western blot using different sources of antibody did not present a specific ARHGAP30 band at the expected size. Nevertheless, we have decided to examine ARHGAP30 at protein level in future studies. By pathway enrichment analysis, we found that the most prominent changes occurred in the extracellular matrix (ECM)-remodeling pathway including TIMP metallopeptidase inhibitor 3 (TIMP3) and GPRIN family member 3 (GPRIN3). Changes in these genes may explain, at least in part, the inhibitory effect of vitamin C on A2058 cell migration and anchorage-independent growth.

**Table 1:** **Differentially expressed genes in A2058 cells used by vitamin C treatment**

**No**	**Symbol**	**Gene**	**RNA-seq (DESeq)**		**qRT-PCR**	
			**Fold**	***P*** **value**	**Fold**	***P*** **value**
1	ARGHAP30	Rho GTPase activating protein 30	15.2	4.73 × 10^−21^	10.3	2.2 × 10^−5^
2	TRIM63	Tripartite motif containing 63, E3 ubiquitin-protein ligase	5.3	2.15 × 10^−05^	4.3	2.2 × 10^−5^
3	PTPN7	Protein tyrosine phostaphatase, non-receptor type 7	3.8	0.01	2.6	8 × 10
4	SOCS3	Suppressor of cytokine signaling 3	1.9	1 × 10^−4^	2	9 × 10^−3^
5	FAP	Fibroblast activation protein, alpha	1.7	3.43 × 10^−21^	1.6	0.01
6	GPRIN3	GPRIN family member 3	1.7	5 × 10^−5^	2	3 × 10^−4^
7	TIMP3	TIMP metallopeptidase inhibitor 3	1.6	5 × 10^−5^	1.5	0.01
8	MAGEA4	Melanoma antigen family A	1.6	5 × 10^−5^	1.8	1 × 10^−4^
9	SERPINE2	Serpin, peptidase inhibitaor clade E, member 2	0.67	2.5 × 10^−4^	0.79	0.01
10	SLITRK2	SLIT and NTRK-like family member 2)	0.49	1 × 10^−4^	0.66	2 × 10^−3^

## Discussion

Recent discoveries of the epigenetic abnormalities in cancer provide unprecedented opportunities to reprogram malignant cancer cells and alter them toward healthy cells by targeting related enzymes that are responsible for the epigenetic modifications [21]. The conversion of 5mC to 5hmC requires a catalytic complex that is at least composed of the TET enzymes, the co-substrate 2-oxoglutarate, and the cofactor Fe^2+^. We, for the first time, reported that vitamin C is an additional cofactor for TETs to generate 5hmC. This was subsequently verified by three other groups and is now widely accepted [13-17]. Interestingly, the loss of 5hmC has been identified as a novel hallmark for most, if not all, types of cancer [1-4]. Various mechanisms may underlie 5hmC depletion in cancer such as mutations in TETs and IDHs [1,22] and thus decreased expression of TETs and IDHs [3]. Published studies have shown that overexpressing TET2 in melanoma cells and TET1 in breast cancer cells increases their 5hmC content and decreases their malignancy *in vitro* and *in vivo* [3,23]. However, it might not be feasible to overexpress TETs in cancer patients as a therapeutic option. This study attempted to identify alternative means that may rebuild the 5hmC content in malignant cells as a potential treatment for cancer.

Contrary to what one might expect, more than 7% of the US population (>20 million individuals) is estimated to be deficient in vitamin C (concentrations <11.4 μM in plasma) [24,25]. Further, the turnover rate of vitamin C appears quite rapid, suggesting that the number of people with short-term vitamin C deficiency could be even higher [26]. Possibly due to the fact that it is nearly impossible to quantitatively control dietary vitamin C consumption in human subjects, an epidemiological link between vitamin C deficiency and the incidence of melanoma has yet been published.

Interestingly, recurrent mutations in the splicing factor SF3B1 have been identified in certain types of melanoma [27]. The mutant SF3B1 causes a truncated, most likely nonfunctional, SVCT2 that can result in intracellular deficiency of vitamin C in cancer cells [28]. Besides a lower expression of TETs in melanoma cells as verified in this research, we also found that the expression of SVCTs was also significantly decreased especially in metastatic melanoma cell lines. This result suggests that a local deficiency of vitamin C, due to less transporters in metastatic melanoma cells, could also be responsible for the loss of 5hmC in metastatic melanoma.

Based on the recent findings, including ours, [13-17] we sought to test whether vitamin C could be an alternative of TETs overexpression to restore 5hmC in cultured melanoma cells. Distinct from previous studies of using pharmacological concentrations, we chose vitamin C at physiological concentrations, which can be conveniently reached by diet and dietary supplements, to treat cells. We showed that vitamin C treatment indeed reestablished 5hmC global content toward that in healthy melanocytes. Interestingly, higher concentration of vitamin C did not cause an even higher level of 5hmC in melanoma cells, indicating that vitamin C at physiological levels can satisfy the need of TETs, though at low expression level, and maximize their enzymatic activity to generate 5hmC in melanoma cells.

In addition to the changes in 5hmC content, vitamin C at physiological concentrations also caused phenotypic changes in cultured melanoma cells. *In vitro* assays showed that vitamin C (0.1 mM) treatment decreased the malignant phenotype of A2058 cells by inhibiting cell migration and anchorage-independent growth, while exerting no obvious effect on proliferation rate. Published studies have shown that vitamin C depletion increases the growth and metastasis of melanoma cells in L-gulonolactone oxidase-knockout (Gulo^−/−^) mice that mimic the human inability to synthesize ascorbate [29]. Further, supplementation of vitamin C could inhibit the growth and metastasis of melanoma cells in Gulo^−/−^ mice [30]. These results suggest that vitamin C treatment could decrease the malignant phenotype of melanoma *in vitro* and *in vivo*.

Consistent with the dual function of TETs and the consequent 5hmC on transcription regulation [31], vitamin C-induced 5hmC also correlated with bi-directional effects on transcription, although the most significantly altered transcripts were up-regulated. An ideal study will be an integrated analysis of RNA-seq and sequencing 5hmC in the genome of melanoma cells after vitamin C treatment. However, due to limits in financial support, a detailed 5hmC profiling by high-throughput sequencing can only be conducted in the future. Nevertheless, this research has successfully captured the most significant genes sensitive to vitamin C treatment in A2058 cells.

The most predominant change occurs in the ARHGAP30 gene, of which the expression is increased more than tenfold after vitamin C treatment. Interestingly, not so much is known about the function of this gene. However, a recent report showed that ARHGAP30 is required for acetylation and functional activation in colorectal cancer. A low level of ARHGAP30 expression also is associated with poor survival of CRC patients [32]. A dramatic increase in ARHGAP30 expression caused by vitamin C can enhance the tumor suppressor activity of p53, further decreasing the malignancy. Thus, this study primarily links vitamin C to P53 in cancer treatment.

Furthermore, ECM remodeling appeared to be the primary pathway regulated by vitamin C. Other than GPRIN3, the most notable gene in this pathway is TIMP metallopeptidase inhibitor 3 (TIMP-3). Interestingly, overexpressing TET1 has been shown to activate TIMPs expression in breast cancer cells [23]. The mechanisms of how these altered genes contribute to the decreased malignancy of A2058 cells remain unclear and require further examination in the future.

Astonishingly, vitamin C is often absent in the formulation of the majority of media used for culturing cancer cells [33]. This suggests that the critical regulatory role of vitamin C in the cancer epigenome might have been overlooked in previous cell-based studies. On the other hand, immunocompromised or genetically modified mice have been overwhelmingly used in cancer research. However, unlike humans, mice can synthesize vitamin C *de novo*. This implicates that previous mice-based studies might have ignored the effect of variation in vitamin C availability on the cancer epigenome.

In addition to restoring 5hmC in melanoma cells as described above, vitamin C at pharmacological doses also have been shown to inhibit the activity of DNA methyltransferases and to alter the expression of many miRNAs [34]. Besides these epigenetic pathways, the toxicity of vitamin C at mM ranges is also through reactive oxygen species pathways [35]. Taken together, vitamin C, a safe and well-tolerated micronutrient, needs to be further evaluated in melanoma treatment.

## Conclusions

Our study demonstrates that treatment of vitamin C at a physiological concentration can decrease the malignant phenotype of melanoma cells *in vitro* by partially reestablishing the global content of 5hmC and the consequent alteration in the transcriptome. These results suggest that vitamin C could be a potential epigenetic treatment for melanoma and perhaps other types of cancer.

## Methods

### Cell culture and treatments

Melanocyte line (FOM-113), derived from a healthy human subject, and melanoma cell lines derived from radial growth phase (RGP, SBcl2 and WM35) and vertical growth phase (VGP, WM278 and WM3248) as well as metastatic phase (C8161) were gifts from Dr. M. Herlyn (The Wistar Institute). Another line of metastatic melanoma (A2058) was purchased from ATCC. Cells were cultured in the media as described [33]. The media used to maintain these cell lines did not contain any vitamin C in their formulas. After seeding in 6-well plates for 24 h, cells were treated with vitamin C (L-ascorbic acid, Sigma-Aldrich, St Louis, MO, USA) at different concentrations for varying durations. Each treatment group consisted of three wells for every experiment. Each experiment was repeated at least three times.

### Quantitative real-time RT-PCR

RNA was extracted from cultured melanocytes and various melanoma cells using RNeasy kits (Qiagen, Hilden Germany). A nanodrop 8000 photospectrometer was used to measure the yield of RNA extraction. The SuperScript III First-Strand Synthesis System (Invitrogen, Carlsbad, CA, USA) was used for reverse transcription (RT) according to the manufacturer’s instructions. Quantitative real-time RT-PCR (qRT-PCR) was performed in triplicate on a Roche LightCycler 480. The 10-μl reaction contains SYBR Green master mix, primers, and diluted cDNA (100 ng). All primers were designed to span introns (Table 2). The transcript amplification results were analyzed with the LightCycler 480 software, and all values were normalized to the levels of the GAPDH using the 2^-(ΔΔCt)^ method. Statistical significance of differences in expression levels between melanocytes and different melanoma cell lines or between A2058 cells treated with or without vitamin C were assessed by Student *t* test, at *α* = 0.05.

**Table 2:** **Primers for quantitative real-time RT-PCR**

**Gene**	**Forwards (5’ → 3’)**	**Reverse (5’ → 3’)**
TET1	AATGGAAGCACTGTGGTTTG	ACATGGAGCTGCTCATCTTG
TET2	AATGGCAGCACATTGGTATG	AGCTTCCACACTCCCAAACT
TET3	GAGGAGCGGTATGGAGAGAA	AGTAGCTTCTCCTCCAGCGT
SVCT1	TCATCCTCCTCTCCCAGTACCT	AGAGCAGCCACACGGTCAT
SVCT2	TCTTTGTGCTTGGATTTTCGAT	ACGTTCAACACTTGATCGATTC
ARGHAP30	GTGCCCCAGGTGCTAAAGAG	CTTGGAGGTAAACATCCCGAC
TRIM63	GCTTTGAGAACATGGACTTCTTT	CTTCTGTGGACTCTTCCTCTTC
PTPN7	GGGAGGTCACCCTACACTTTC	TGGTCTTGTATCGGTCCTTGG
SOCS3	GGAGACTTCGATTCGGGACC	GAAACTTGCTGTGGGTGACC
FAP	CAAAGGCTGGAGCTAAGAATCC	ACTGCAAACATACTCGTTCATCA
GPRIN3	ATGGGGACTGTACCTGACCC	GGTGGTCTCATGCTCACAAAC
TIMP3	CGGTATCACCTGGGTTG	GTAGCCAGGGTAACCGAAA
MAGEA4	AGGAGCACCAAGGAGAAGAT	GCTTGCAGTGCTGACTCTT
SERPINE4	CTCGCCATGGTGATGAGATAC	CCTCACACTGGAACACATCTT
SLITRK2	CCTGGAAGCAGCGTCTTATT	CCTACTAAATCCTGCCCATCTC

### Dot-blot assay

Genomic DNA was extracted from cultured melanocytes and various melanoma cells using QIAamp DNA mini kits (Qiagen, Hilden Germany) according to the manufacturer’s instructions. A Qubit Fluorometer (Life Technology, Foster City, CA, USA) was used to quantify the concentration of DNA. The dot-blot procedure followed the published methods as conducted in our previous studies [13]. Briefly, DNA samples were diluted with 2 N NaOH and 10 mM Tris · Cl and pH 8.5 then loaded on a Hybond N+ nylon membrane (GE Health, Piscataway, NJ, USA) using a 96-well dot-blot apparatus (Bio-Rad, Richmond, CA, USA). After baking at 80°C for 30 min and being blocked by 5% non-fat milk for 1 h at room temperature, the membrane was incubated in a polyclonal anti-5hmC antibody (Active Motif, Carlsbad, CA, USA; #39769, 1:10,000) at 4°C overnight. 5hmC was visualized by chemiluminescence. The densities of the dots were captured by AlphaImager. To ensure equal loading, the membrane was stained with methylene blue post-immunoblotting. Statistical significance of differences in 5hmC content between different treatments were assessed by Student *t* test at *α* = 0.05.

### MTT assay

Cell growth was measured using MTT cell proliferation kits (BioVision Technologies, Exton, PA, USA) according to the manufacturer’s instruction. Briefly, 5 × 10^3^ cells/well were cultured in 96-well plates and cultured in the medium supplemented with or without vitamin C as indicated in individual experiments. Samples were assayed in six replicates and these experiments were repeated three times.

### Cell migration assay

Tumor cell migration was tested in transwell using BD Falcon FluoroBlok™ Systems with an 8-μm porous membrane insert (BD Biosciences, San Jose, CA, USA). A total of 1 × 10^4^ A2058 melanoma cells were suspended in a 0.3-mL serum-free medium and seeded in 8-μm pore Fluoroblok inserts and left to migrate toward the lower chamber containing 0.7 mL of tumor medium with 1% FBS. Wells were divided into two groups (three wells/group) as follows: vitamin C (+) and vitamin C (−) groups. In the vitamin C (+) group, vitamin C (0.1 mM) was supplemented in both the insert and the low chamber. Cells were cultured at 37°C for 16 h. Migrated cells were counted using an Olympus TH4-100 fluorescence microscope connected to a DP-70 Olympus digital camera (Olympus, Tokyo, Japan).

### Colony formation assay

Tumor cell colony formation in soft agar was carried out as described [36]. Briefly, 3 × 10 A2058 melanoma cells per well were embedded into 0.33% agar gel containing FBS in 6-well plates pre-coated with 0.5% of agar solution in triplicate and covered with tumor medium supplemented with or without vitamin C (0.01 or 0.1 mM) or as indicated in the individual experiment. Medium was replaced every other day. Colonies (defined as a minimum of four cells) were counted after 15 days of incubation.

### RNA-seq

A2058 cells cultured in 6-well plates were treated with or without vitamin C (0.1 mM) for 48 h. Medium was changed daily before each treatment to avoid the accumulation of vitamin C in the medium. Total RNA was then extracted from these cells. A Bioanalyzer 2000 (Agilent, Palo Alto, CA, USA) was used to monitor the quality of RNA. All samples’ RNA integrity numbers (RIN) were above nine (data not shown). The whole transcriptome sequencing was carried out at the Sequencing Core of John P. Hussman Institute of Human Genomics at the University of Miami. Briefly, after ribosome RNA (rRNA) was depleted, sequencing libraries were constructed following the standard Illumina protocols and were subsequently processed by a Hiseq2500 (Illumina, San Diego, CA, USA) sequencing system (200 bp paired-end reads, four samples per lane). Raw read data was first run through quality control metrics using FastQC (http: //www.bioinformatics.babraham.ac.uk/projects/fastqc/). After quality control was checked, sequence reads were aligned using the STAR aligner [37]. The aligned reads from the STAR aligner was then run through HTseq for transcript quantification against the GENCODE v19.gtf file [38]. After all features were quantified, the data was then run through three different differential expression calculators in EdgeR, DESeq, and BaySeq [39-41]. The intersection of the three methods were taken and transformed into a list of the final differentially expressed features. Differentially expressed features were determined by cutoff adjusted *P* values of 0.05 across all three methods. That final list was then put through gene ontology annotation using the BiNGO added into Cytoscape v 3.1.1 [42]. The list was also run through GeneGO for pathway enrichment (https://www.portal.genego.com).

### Statistical analysis

All data were normalized by inner controls, such as GAPDH expression level. Data was presented as mean ± SD. Statistically significant changes among treatments were assessed by Student *t* tests at *α* = 0.05.

## Abbreviations

5hmC: 5-hydroxymethylcytosine
5mC: 5-methylcytosine
ARHGAP30: rho GTPase-activating protein 30
ECM: Extracellular matrix
GPRIN3: GPRIN family member 3
Gulo: L-gulonolactone oxidase
IDH: Isocitrate dehydrogenases
RGP: Radial growth phase
SVCT: Sodium-dependent vitamin C transporters
TET: Ten-eleven translocation family dioxygenases
TIMP3: TIMP metallopeptidase inhibitor 3
VGP: Vertical growth phase
